# Food Acquisition Coping Strategies Vary Based on Food Security Among University Students

**DOI:** 10.1016/j.cdnut.2024.104529

**Published:** 2024-12-16

**Authors:** Emily Sklar, Gwen M Chodur, Leslie Kemp, Deborah S Fetter, Rachel E Scherr

**Affiliations:** 1 Department of Nutrition, University of California, Davis, CA, United States; 2 Aggie Compass Basic Needs Resource Center, Davis, CA, United States; 3 The Family, Interiors, Nutrition & Apparel (FINA) Department, San Francisco State University, San Francisco, CA, United States; 4 Scherr Nutrition Science Consulting, LLC, San Francisco, CA, United States

**Keywords:** food insecurity, coping mechanisms, USDA AFSSM, college students, food access barriers

## Abstract

**Background:**

Food insecurity on college campuses is a pressing issue, yet the ways in which students manage challenges and disruptions to their food security status (FSS) are poorly understood.

**Objectives:**

The objective of this study was to examine knowledge of food insecurity as a concept, evaluate FSS, identify food acquisition-related behaviors, and determine whether these behaviors differ among FSS.

**Methods:**

University students at increased risk of experiencing food insecurity (*n* = 43) were recruited for this mixed-methods study. Participants were surveyed about their FSS, coping strategies, and use of food access resources. Subsequent interviews occurred to evaluate their understanding of food insecurity as a concept and related food acquisition behaviors. The total number of coping strategies and food access resources used were quantified, and composite variables were created. Differences based on FSS classification were examined through regression analyses. Quantitative and qualitative data were integrated using concurrent triangulation.

**Results:**

Despite targeted recruitment efforts, 76% of participants were classified as food secure by the United States Department of Agriculture (USDA) Adult Food Security Survey Module (AFSSM). Participants were able to define food insecurity and identify circumstances that could contribute to an individual becoming food insecure. However, many participants described experiences that suggested the USDA AFSSM may not accurately capture students' true FSS. Most individuals faced significant challenges in maintaining food security. Participants used a series of coping strategies and food access resources to maintain or improve their food security, regardless of FSS, which included couponing, strategizing when food was low, and sharing food with housemates. Additionally, participants expressed concerns that their use of food access resources may deprive others with greater need.

**Conclusions:**

Results from this study shed insight on the complexities of food insecurity in the university setting, providing useful data to inform the development of better programs, outreach, and evaluation tools that encapsulate the many unique factors that make up FSS for students.

## Introduction

Food insecurity is a major issue linked to significant negative physical, behavioral, and mental consequences [[1], [2], [3]]. Food insecurity refers to a situation in which an individual has limited or uncertain access to adequate food or food of adequate quality [4]. As of 2023, the prevalence of households experiencing food insecurity is 13.5% within the United States [5]. However, the prevalence of food insecurity varies within the population, with some population groups experiencing rates above the national average [6].

College students are one such population at an increased risk of food insecurity because of the financial burden of tuition and other school-related expenses, rising cost of living, and limited time to hold a full- or part-time job [7]. Estimates of college student food insecurity status vary, but reports from 2020 have estimated that nationally, ∼30% to 42% of college students report experiencing low or very low food security [[8], [9], [10]].

A robust body of evidence has demonstrated associations between food insecurity and negative health-related behaviors on college campuses [11,12]. Multiple studies report that college students experiencing food insecurity were more likely to perceive their health as “fair” or “poor” compared with students who are food secure [13,14]. In one 2022 study conducted within the University of California (UC) system, food insecurity was associated with higher BMI and an increased likelihood of poor perceived health status [15]. Research has also shown that college students experiencing food insecurity tend to have lower fruit and vegetable consumption and higher added sugar intake than students who are food secure [16,17]. Additionally, poorer mental health and increased depression-related cognitive impairments have been associated with food insecurity in college student populations [18,19]. In one study, college students facing food insecurity had 3.18 times greater odds of experiencing depression and 4.35 times greater odds of experiencing anxiety compared with students who are food secure [13].

Particularly concerning for college students are the negative associations between food insecurity and academic performance [20]. College students struggling with food insecurity were more likely to drop out, withdraw from courses, and have lower grade point averages (GPAs) compared with students with food security [[21], [22], [23]]. A study within the 10-campus UC system reported that students with food security were more likely to hold an “A” cumulative grade average (51%) compared with students with food insecurity (30%) [24].

Although the deleterious effects associated with food insecurity in this population are well documented, there is less known about the myriad of contexts in which food insecurity occurs in college students and how students navigate challenges in food access. Food acquisition behaviors, commonly referred to as coping strategies, can serve as one mechanism to understand how college students address food insecurity [25]. Techniques used to obtain food, decrease barriers to food acquisition, and reduce financial burden serve as coping strategies for college students facing episodic and persistent food insecurity [26]. Coping strategies noted within the literature include purchasing cheap food, stretching food when low, and eating less healthy meals in order to eat more [27,28]. In addition, these behaviors are often associated with disordered eating behaviors and/or diminished diet quality [[29], [30], [31]]. Despite the impact coping strategies can have on the individual, these behaviors are not typically considered when classifying food security status (FSS) [27,32]. Thus, an exploration of student experiences and the methods that students use to cope with food insecurity is vital to designing effective interventions, evaluation tools, and resources to mitigate the impacts of food insecurity in this vulnerable population.

The purpose of this study was to assess knowledge about food insecurity as a concept, evaluate their FSS, identify food acquisition-related coping strategies, and determine if such behaviors differ according to FSS. To gain a deeper understanding of how FSS influences food acquisition-related behaviors of college students at risk for food insecurity, a mixed-methods analysis examining differences in experiences, food acquisition behaviors, and food access use was conducted. It was hypothesized that college students with low and very low food security would use more coping strategies and food access resources to maintain food security than their peers with marginal and high FSS.

## Methods

### Study design

This mixed-methods study employed a 44-item questionnaire administered to students during the summer of 2021 to assess participants’ use of coping strategies and food access resources. The Adult Food Security Survey Module (AFSSM) was used to classify participants’ FSS. In-depth interviews were conducted to further assess participants’ understanding of food security as a concept, their personal FSS, and their use of coping strategies and food access resources. Qualitative data were collected to complement the quantitative results and provide a deeper understanding of student experiences. The triangulation of quantitative and qualitative data allowed for exploration of participants’ perceptions of their own FSS, circumstances that could lead someone to becoming food insecure, their use of coping strategies, and use of food access resources.

### Participants and recruitment

A purposive sampling method [33] was used to identify subpopulations at heightened risk for food insecurity based on previous research, including nontraditional students, transfer students, students who previously used on-campus food access resources, students enrolled in CalFresh, graduate students, and/or first-generation students [29, [34], [35], [36], [37]]. Participants were recruited from the UC, Davis (a suburban, public land-grant university) between May and August 2021 through broad and targeted online advertisements administered to various campus/student organizations by email, including but not limited to the Transfer and Re-entry Center, on-campus family housing, and the on-campus Aggie Compass Basic Needs Center. These locations were selected to recruit individuals at a higher risk of facing food insecurity. Recruitment emails contained information about the study and a web link to complete a 17-item screening questionnaire to determine eligibility. To reduce volunteer bias, advertisements included limited information on the study background and used neutral language, such as FSS, rather than the term “food insecurity.” Individuals were eligible to participate if they were aged ≥18 y, had been enrolled at the UC, Davis in the academic quarter prior to the study period, and had access to a computer with Internet. Individuals who had been on a campus meal plan in the previous quarter were excluded, as they had a more predictable source of food.

### Quantitative methods

#### Questionnaire

Eligible participants were invited to complete a questionnaire administered through Qualtrics. Prior to beginning the questionnaire, consent was collected for participation and retrieval of institutional data. The 44-item survey tool incorporated several validated quantitative questionnaires to investigate aspects of the student experience. The National Food Access and COVID research Team Questionnaire was used to evaluate participants’ usual sources of food and strategies for affording or acquiring food [38]. Pre- and post-COVID questions on shopping behaviors, coping strategies, and food access were employed to assess food acquisition behaviors within the population. These items, previously validated in the general adult population using Cronbach’s α and factor analysis, specifically focused on how participants obtained food [38]. Examples of strategies included accepting food from friends, borrowing money from friends/family, and purchasing food on credit. Additionally, participants were asked if they currently, previously, or planned to use these strategies in the future [16].

Using an author-generated list, participants were asked if they used food access resources, such as CalFresh, on-campus food access programs, community food pantries, and/or state or federal-funded food assistance programs (Supplemental Nutrition Access Program [SNAP], Supplemental nutrition program for Women, Infants, and Children [39], or Food Distribution Program on Indian Reservations [FDPIR]). As well as campus-specific food distribution programs, including the UC Davis Aggie Compass Basic Needs Center, the Associated Students, University of California, Davis student pantry, the Graduate Student Association pantry, and a free fruit and vegetable pop-up. FSS over the last 30 d was measured using the 10-item USDA AFSSM [10]. Self-reported demographic information was collected, including first-generation status (participants whose parents did not complete a 4-y degree) [40]. Participants were also asked if they were a nontraditional students, as these students tend to be at higher risk for experiencing food insecurity [41,42]. This group includes individuals who delayed enrollment immediately following high school, attend college part-time, work ≥35 h/wk while enrolled, are financially independent, have dependents other than a spouse, do not have a high school diploma, and/or are > 25 y upon undergraduate entry [43]. Participants responded to questions about paid and unpaid employment, as well as financially-related behaviors, such as credit card use, family support, and financial aid.

The questionnaire, interview guide, and consent procedure were approved as exempt research by the UC, Davis Institutional Review Board. Written and oral consent was obtained for participation and recording of in-depth interviews.

Institutional data for participant demographics (race/ethnicity, sex, academic class standing), admission type (freshman, transfer, or graduate), and cumulative GPA were retrieved from the Center for Student Affairs Assessment. The institutional data were combined with questionnaire responses to complete the analytic data set.

#### Data analysis

Eight items related to coping strategies from the National Food Access and COVID research Team Questionnaire were included to understand food acquisition behaviors among participants [38]. Participants’ responses to the question of coping strategies used to afford food were coded as 0 (“Not using now” or “Prefer not to answer”) or 1 (“Using now”). A composite variable for total strategies was created by summing the number of affirmative responses from each participant and ranged from 0 to 8. Participants’ responses to questions regarding use of food access resources within the last month were coded as 0 (not selected) or 1 (used). A composite variable for number of food access resources was created by summing the number of resources indicated by each participant (ranging from 0–3).

Using the AFSSM coding scale, participants were classified into 1 of 4 food security groups: high (0 affirmative responses), marginal (1–2 affirmative responses), low (3–5 affirmative responses), or very low (≥6 affirmative responses).

Participants were classified as having received need-based financial aid if they reported currently receiving or having received any of the following: Pell Grant (federal need-based aid award), CalGrant (state need-based aid award), or a Blue and Gold Grant (campus need-based aid award).

#### Statistical analysis

Frequency statistics were calculated for descriptive characteristics of the complete sample. Tetrachoric correlations were conducted to test agreement between responses to questionnaire items and qualitative experiences measuring FSS, food access use, and food acquisition strategies. Comparisons by FSS were made for each coping strategy used to afford food and for use of each food access resource by chi square tests. Incidence rate ratios were calculated for each of the cumulative variables using Poisson regressions with the composite variable as the dependent variable and FSS as the independent variable with adjustment for covariates. Sociodemographic characteristics, including first-generation status, nontraditional student status, sex, race/ethnicity, academic class standing, admission type, GPA, and receipt of need-based aid, were tested as covariates because of their association with food insecurity in college student populations [34,36,37]. Student level (graduate or undergraduate) was tested as a covariate and found to be not significant; therefore, data were analyzed as a single sample. All covariates were tested, but covariates with a *P* > 0.20 were excluded from final adjusted models for parsimony. Final models included cumulative GPA and a history of receiving need-based financial aid. Statistical significance was set to *P* < 0.05. All analysis was conducted using Stata SE v16.1 (StataCorp).

### Qualitative methods

#### Interviews

After completion of the questionnaire, participants were emailed to schedule an interview for the qualitative portion of the study. Recruitment continued until key themes within the qualitative interviews achieved saturation [44]. Using a deductive approach, an in-depth interview guide was developed in tandem with the quantitative questionnaire to provide context for the survey responses and offer deeper insights into participant experiences (Supplemental File 1). The guide was established under a phenomenology framework and designed to be adaptable to participants’ responses to further explore their experiences and specific coping strategy use.

Participants were asked a series of questions about food insecurity as a concept, circumstances that could lead to someone becoming food insecure, their personal FSS, food acquisition behaviors, and use of food access resources. Two basic needs experts (LK and GC) familiar with on-campus resources and food insecurity within the college student population reviewed the interview guide.

One-on-one 25 to 60 min interviews were conducted over Zoom by 2 female student analysts (ES and GC) trained in qualitative interviewing techniques. No repeat interviews were carried out. Interviewers and participants had no prior acquaintance, and participants were not informed about the specific purpose or context of the research study to avoid potential bias. Oral consent was obtained, and participants agreed to be recorded as part of the consent procedure. Zoom’s transcription functionality was used for the initial transcription and compared against the video recording by a research assistant to ensure accuracy. Transcripts were not returned to participants for comment or correction.

#### Data analysis

Identifying information within transcripts was redacted prior to analysis by the research team. The same 2 analysts (ES and GC) inductively content-coded 2 transcripts individually line-by-line before meeting to compare and discuss findings. From this, a codebook was developed with systematic instructions and examples to ensure consistency between multiple analysts [45]. The proposed codebook was applied to the previously coded transcripts by the same 2 analysts, who then met to discuss findings and reconcile coding [46,47]. A pooled Kappa score was calculated based on interrater agreement, yielding a score of 0.77 or “fair” agreement under established indices [48,49]. The codebook was further refined, and both analysts coded 2 more transcripts. A second pooled Kappa score was calculated for the reconciled coding, yielding a score of 0.92, demonstrating “excellent” agreement under established indices [48]. The analytic codebook was based on the consensus that emerged from this meeting and was composed of a coding frame of 11 coding families and 113 total codes. Each remaining transcript was double-coded by the same 2 analysts. On completion of coding, data were extracted and analyzed for key themes to be compared with quantitative results for interpretation. All coding and analysis were conducted using Atlas.ti (Atlas.ti) software.

### Data triangulation

This study used a concurrent triangulation design to compare qualitative and quantitative findings [50,51]. Qualitative and quantitative analysis occurred concurrently, with emergent qualitative themes being compared with quantitative findings to identify areas of convergence and divergence between the qualitative and quantitative findings. As an example, when an unanticipated finding regarding the use of food access resources by participants at various food security classifications emerged from quantitative analyses, qualitative findings were examined to better understand why expected differences were not observed. An examination of the qualitative data demonstrated utilization of coping strategies beyond using food access resources within all food security classifications, which led to a quantitative analysis of the totality of coping strategies used by students.

## Results

**FIGURE 1:**
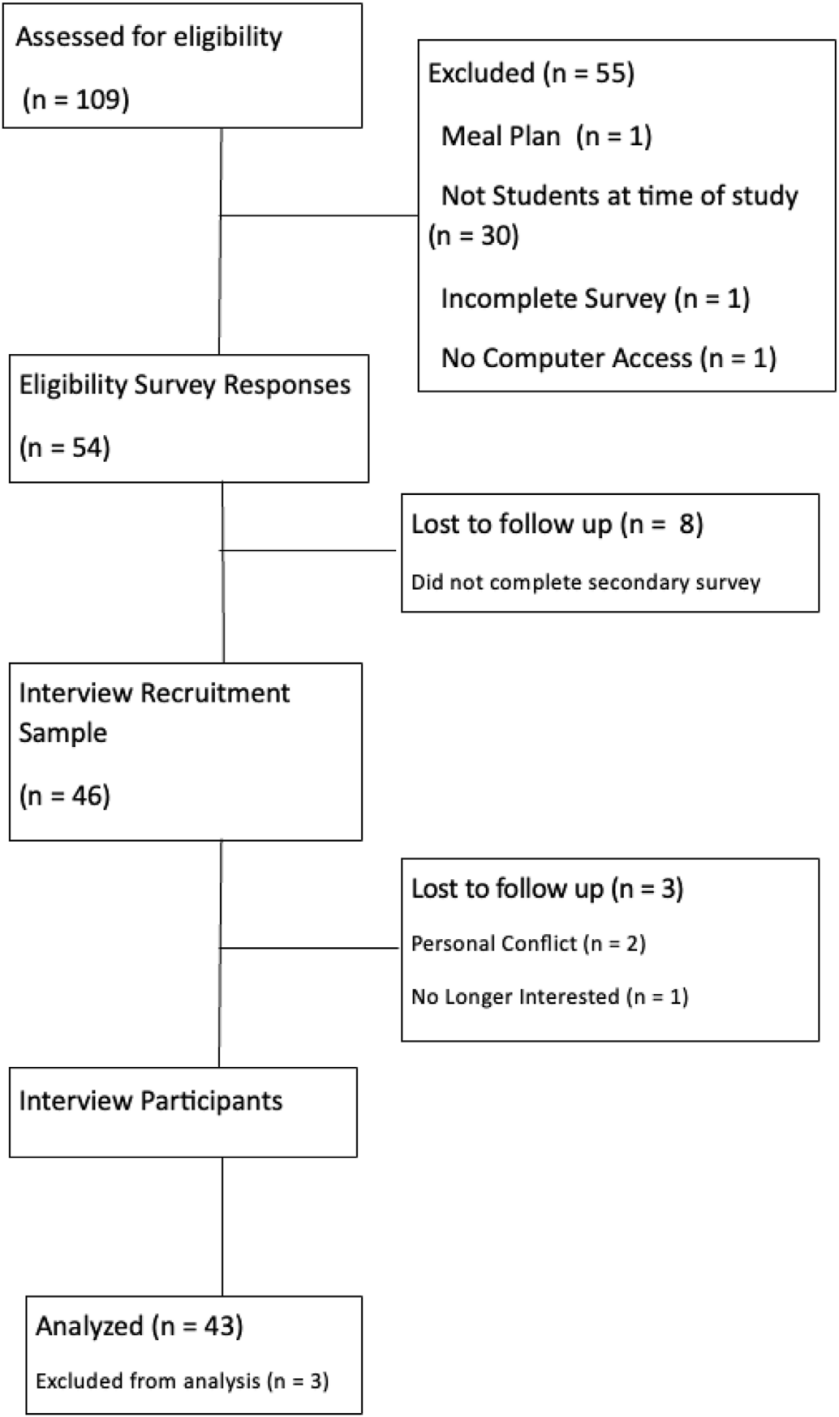
CONSORT diagram.

Forty-three participants completed the questionnaire and the qualitative interview (Figure 1). The majority of participants identified as female (77%) undergraduate students (79%) who held some form of paid employment (60%). Two thirds of the population lived in off-campus housing, and 37% reported that they had received need-based aid at some point during their enrollment (Table 1). Based on the AFSSM, approximately half of the participants were classified as having high food security (51%), 11 participants were classified as having marginal food security (25%), 5 participants were classified as having low food security (12%), and 5 participants were classified as having very low food security (12%) (Table 1). Four main themes were derived from the data, including a high level of knowledge about food insecurity, various college student-specific challenges in maintaining food security, a varied repertoire of coping strategies used across all categorizations of FSS, and conflicted feelings about the utilization of food access resources.

**TABLE 1:** Sociodemographic characteristics of participants (*n* = 43) who completed both phases of the study.

Characteristics	Total (*n* = 43)
Sex, n (%)	
Male	9 (21.4)
Female	33 (78.6)
Class standing, n (%)	
Undergraduate	34 (79.1)
Freshman	3 (7.4)
Sophomore	8 (19.1)
Junior	8 (19.1)
Senior	15 (35.7)
Graduate	9 (20.9)
GPA, mean±SD^1^	3.55±0.41
First-generation, n (%)	17 (39.5)
Nontraditional student, n (%)	11 (25.6)
Received need-based aid, n (%)	16 (37.2)
Race and ethnic status, n (%)^1^	
White	11 (26.2)
Black	1 (2.4)
American Indian/Alaska Native	3 (7.1)
Chinese American/Chinese	9 (21.4)
East Indian/Pakistani	3 (7.1)
Filipino/Filipino-American	2 (4.8)
Vietnamese	1 (2.4)
Mexican-American/Mexican/ Chicano/a/x	9 (21.4)
Latino/a/x/ Other Spanish	3 (7.4)
Paid employment, n (%)	26 (60.5)
Weekly hours, mean ± SD	21.3±12.5
Unpaid employment, n (%)	15 (34.8)
Weekly hours, mean ± SD	10.2±7.1
Food Security Status, n (%)	
Food Security	22 (51.2)
Marginal Food Security	11 (25.6)
Low Food Security	5 (11.6)
Very Low Food Security	5 (11.6)

1 *n* = 42; institutional data could not be retrieved for one individual.

### Knowledge of food insecurity

The majority of participants displayed a high level of knowledge about how food insecurity affects both the quantity of food that someone can acquire and the quality of the food available. As defined by one participant, “Food insecurity is the inability or the stress of not knowing where…if you’re going to be able to have enough food for the next coming days, or also not able to have a healthy source of food. If you have to rely on cheap food or fast food, I would say, [that is] also food insecurity” (Interview 38, marginal food security). Another participant defined food insecurity as “an inability to access or afford the foods that can sustain a well-balanced diet. I also think that would maybe also include just foods that bring happiness and fulfillment” (Interview 37, very low food security). Additionally, participants were able to identify various circumstances that could lead to someone becoming food insecure, as demonstrated by the response, “Definitely just income and not knowing if you’re going to have enough money is one of the main things. […] How close is the nearest market to you? Are you able to just go out quickly to get some groceries to make a meal?” (Interview 38, marginal food security).

Although participants were able to identify circumstances that could cause someone to be food insecure, their perception of food insecurity differed when speaking about their own experiences. Whereas participants were largely able to define food security and 76.6% of the sample were classified as having high or marginal food security, many participants described experiences of limited food access. “I don’t look for help when I need to, and that makes it kind of hard for me to get out of that constant cycle of not knowing whether I’m going to have food the next day” (Interview 35, marginal food security). As part of the in-depth interview, participants were asked if they agreed with their FSS as classified by the AFSSM. Participants often agreed with their classification yet went on to discuss experiences that may be indicative of more precarious food security than their classification may suggest. There were also notable examples of participants who did not agree with their classification, such as one student who said “I honestly don’t know; I still have instant noodles. It’s not like I’m starving every day…No, I don’t agree [with AFSSM classification]” (Interview 15, very low food security). Considering these observations, inconsistencies in expected response patterns within survey responses were identified. One component of the food acquisition strategies question was identified as seeking similar information to a question within the AFSSM (food access strategies: “Which of the following strategies are you using now to afford food: stretch the food I have by eating less” and AFSSM: “In the last month, did you ever cut the size of your meals or skip meals because there wasn’t enough money for food?”). The tetrachoric correlation between responses to these 2 items was r_tet_ = 0.72; *P* = 0.005.

### Challenges to maintaining food security

All participants, regardless of FSS, recounted challenges to maintaining food security in college. Participants shared experiences in which access, finances, and time impacted their ability to acquire food. Many participants reported facing common challenges, including competing financial obligations and rising costs of living. One individual expressed, “Everything’s harder out here; the cost of living is harder, and the price that they pay graduate students in California is not enough to thrive out here” (Interview 40, high food security). Several individuals discussed the challenge of affording groceries: “I live nearby Trader Joe’s, so that has been a very convenient place, but not all of their products are going to work for me in terms of price” (Interview 37, very low food security).

In addition to challenges shared with outside adult populations, participants described challenges specific to the college student experience. One individual discussed the impact of receiving financial aid disbursements once an academic quarter. “The start of the quarter, I find myself buying more frozen foods, fruits and vegetables and [...] towards the end of the quarter, I find myself buying more top ramen just to make it through” (Interview 8, high food security). In addition to the timing of financial aid disbursements, participants identified other challenges that made it difficult to maintain food security, including the cost of attendance, adjustment to independent living, and limited time, as well as other college student-specific challenges, as seen in Table 2.

**TABLE 2:** Challenges to achieving food security identified by *n* = 43 university students participating in indepth interviews.

Challenge	Exemplar quotes
Adjusting to independent living	“It was kind of crazy to go from having meals that were prepared for me all the time to not really having anything, and I was just kind of thrown into it.” - Interview 33, very low food security
	“After I moved out of the dorms and kind of had to buy groceries myself and all that I was kind of like “oh shoot I don't have enough money to buy groceries for myself, and I don't know if I’ll have enough money to buy groceries for this month.” – Interview 1, very low food security
	“it's something [food insecurity] that I mainly experienced in my second year of college, because my first year I was in the dorms and like there were meals like I could just go to the dining commons without like cooking it myself. And, but then like second year came by I moved out of the dorms, and I had to figure out my schedules and fit in like the food into my schedule” – Interview 24, marginal food security
Financial dependence	“My parents barely have enough to pay for rent and house my other siblings. So, I've had to get the money myself working throughout high school, and even now I've had to work in order to pay off the tuition and even then, I still have thousands of dollars in debt.” – Interview 2, very low food security
	“The fact that someone else is supplying basically my necessities in college, there's that feeling of being a burden, because you're there someone else's spending money on your food. So, for me it's I’m literally insecure about spending money that isn't mine.”– Interview 6, high food security
	Since I'm an international student I'm not actually allowed to work in the US, so my parents still pay for my tuition […] I need to be aware of what I am spending, what I am planning, because the cost and the time it takes for them to send the money here. – Interview 15, very low food security
Graduate student status	“When I transitioned into Grad school, we were paid in arrears and so I didn't have a paycheck for a month. And when I transitioned from the grant to GSR this month I'm still trying to figure out where the rest of my money is. It's always like these transition periods, where it's just my money's just in some gray area, and I have to figure out where it is because nobody else is going to do it for me, sort of situation.**”** – Interview 40, high food security
	“During the summer session as a graduate student I don't get much money for my TA positions. It's reduced to two thirds or even maybe half of what I normally get paid, which is not a lot to begin with.” – Interview 37, very low food security
Financial payout – time of the quarter	“So, after I get my financial aid, I splurge, I get veggies and fruits and I still get some cheaper products that I know will last long, like pantry products. But I would say at the start of my refund I eat more vegetables than at the end of my refund because I progressively have to budget more and more towards the end.” – Interview 20, marginal food security
	“During the summer session as a graduate student I don't get much money for my TA positions. Its reduced to two thirds or even maybe half of what I normally get paid [,,,] I made the mistake of not realizing that for this summer, my pay was going to be reduced,,,] i'm telling myself it's intermittent fasting, but it isn’t.” Interview 39, high food security
Time	The only time I really have the time to cook myself a meal is breakfast because I wake up early just by choice. But like lunch I must rush in between classes to eat something so it's usually leftovers from the previous day, a microwavable meal that I can cook in like five minutes or less or just something else that I can get really fast. And then dinner is about the same because I'm at work, and I only get a 30-minute break which really doesn't go that far, when I'm trying to like to make a meal, so I just do the same thing, a microwavable meal, or something that I could do quickly. – Interview 19, marginal food security
	“I get so consumed with classes all the time that sometimes I even forget, I skip lunch, not even on purpose it's because all the classes are lined up. – Interview 11, marginal food security
	“Sometimes I can be really focused and work and, working on an assignment and then I get hungry and then have to go figure out something to eat and it just distracts me and it's time that I could have been saving if I had snacks or something” – Interview 2, very low food security
Cost of attendance	I know most PhD programs [But some programs] you have to pay out of pocket for it. You might be working 20 hours a week, which would get you above the threshold, because they assume it's a living wage but they're not factoring in that $50,000 in tuition you’re paying. – Interview 28, high food security
	Well, the financial aid pays for a good amount of the tuition, but financial aid also includes loans. So, I guess it helps me get into college and helps me get through college. But it's still a bunch of loans that I have to worry about in the future – Interview 2, very low food security
	I always forget that there are these kind of surprises fees included with the graduate student tuition stuff, so at the beginning you have to pay for something. I still don’t even know what it’s for, but you have to pay like $270... $200 is like a tenth of my paycheck or a little bit more even, so those little surprise things can really get you. – Interview 37, very low food security
	I didn’t realize how many expenses I had. I was like okay it’s just rent and food and transportation, but in reality, it’s say paying for a car, like car insurance, paying for credit card bills, basically paying for extracurricular activities, they all have their membership requirements, and some clubs require you to go to fundraisers and paying for that. Apart from just the basic necessities there’s so many other things you have to pay for, and I didn’t really register that until I tried it for myself, and I was like ‘wow, that’s a lot’ – Interview 11, marginal food security
	It's hard to wrap your head around how something can cost you $50 to $75,000 per year […] this is before health insurance, before all the living costs associated with being in society and you’ve got that extra 50 grand on top of it, compared to everyone else’s starting point – Interview 38, high food security

Participants also discussed the impacts that resulted from their experiences with food insecurity. One participant described the precarity of their FSS during their time as a student: “It’s kind of like undulating waves of food insecurity,” where some months there would be enough money for food, but in other months they resorted to skipping meals to get by (Interview 37, very low food security). Another student reflected, “I think there is just a residual mental health impact with not having had food. That I always feel like ‘oh gosh, like there’s still a little bit of food in my place’… but if it starts to deplete, I start to get panicky over it because I’m afraid” (Interview 36, marginal food security). Others described how their previous experiences with food insecurity led to ongoing anxiety about having enough money to purchase food: “So you have to plan ahead and I’m good at planning ahead, but it’s always the what ifs, and it’s just a relief to look in your bank account and see that money has been added, and not have to worry as much” (Interview 20, marginal food security). Others discussed the feeling of needing to save money and how that led to the continued use of food access resources, like the campus food pantry: “Sometimes I just feel like I should save money, and I would try to check out what free resources there are first and then see what I can grab from there and then buy whatever I can’t find there” (Interview 15, high food security).

### Coping strategies

Nearly all (90.4%) participants reported the current or ongoing use of at least 1 coping strategy to afford food, with the most frequently used strategies including purchasing less expensive food items (74.4%) and accepting food from family and friends (62.7%). Asking family and friends for money to purchase food was one of the least frequently used strategies (18.6%).

There were significant differences in the total number of coping strategies used by FSS. Participants with marginal and very low food security used significantly more coping strategies than participants with high food security and low food security, as shown in Table 3. Participants who were marginally food insecure used 1.57 more coping strategies than participants who had high food security (*P* = 0.03). Participants with very low food security used 1.9 more strategies than participants classified as having high food security (*P* = 0.01). There were no differences in the number of coping strategies used by participants with low food security and those with high food security.

**TABLE 3:** Food access strategies among *n* = 43 university students.

	Total *n* = 43	High Food Security *n* = 22 (51%)	Marginal Food Security *n* = 11 (26%)	Low Food Security *n* = 5 (12%)	Very Low Food Security *n* = 5 (12%)	P-Value
Which of the following strategies are you using now to afford food? n (%)						1
Accept food from friends or family	27 (62.8)	12 (55)	10 (91)	2 (40)	3 (60)	0.14
Borrow money from friends or family	8 (18.6)	3 (14)	4 (36)	0 (0)	1 (20)	0.28
Buy different, cheaper food	32 (74.4)	13 (59)	11 (100)	3 (60)	5 (100)	0.03∗
Buy food on credit	12 (27.9)	4 (18)	4 (36)	2 (40)	2 (40)	0.54
Buy foods that don’t go bad quickly	32 (74.4)	13 (59)	10 (91)	4 (80)	5 (100)	0.11
Get food from a food program	7 (16.3)	3 (14)	2 (18)	0 (0)	5 (100)	0.37
Stretch the food that I have by eating less	15 (34.9)	5 (23)	3 (27)	2 (40)	5 (100)	0.01∗
Rely more on hunting/fishing/foraging/ growing my own food	4 (9.3)	2 (9)	0 (0)	0 (0)	2 (40)	0.07
Total strategies used^2^, mean (SD)	3.19 (0.26)	2.5 (0.33)	4.0 (0.4)	2.6 (0.6)	5.0 (0.55)	0.010∗
Total strategies used, IRR^3^ (SE)		1.00	1.57 (0.33) *P* = 0.034∗	1.00 (0.33) P = 0.992	1.93 (0.49) *P* = 0.009∗	
Total food access resources used, Mean (SD)^4^	1.1 (0.51)	1.1 (0.53)	0.91 (0.30)	1.2 (0.84)	1.2 (0.45)	0.258
Total food access resourced used, IRR (SE)^4^		1.00	0.80 (0.32) *P* = 0.563	1.00 (0.49) *P* = 0.993	1.2 (0.56) *P* = 0.748	

∗ indicates statistically significant findings *P* <0.05.

1 Between group differences measured by chi square test.

2 Variable created by summing the number of affirmative responses to each of the listed strategies.

3 Multivariate Poisson regression including receipt of need-based aid and cumulative GPA as covariates.

4 Food access resources include: The UCD Aggie Compass Basic Needs Center, the undergraduate student association pantry, the Graduate Student Association pantry, free fruit and vegetable pop-up, off-campus community food banks or food distributions, and state or federal food assistance program (CalFresh/SNAP, WIC, FDPIR).

Most participants (93%) reported using at least 1 food access resource within the past month. Moreover, there were no differences in the use of food access programs by FSS across groups (χ^2^ = 11.26; *P* = 0.26) (Table 3).

In-depth interviews identified other coping strategies not captured on the questionnaire, which included skipping meals, strategizing grocery purchases, buying in bulk, and sharing food with housemates (Table 4). One individual noted, “sometimes if my housemates had food in the pantry, I’d be like ‘I’ll buy it when I can or I’ll replace it, can I just use this for like the week?’ and then like next week my deposit should hit and then I can replace it…. [My housemates are] all pretty understanding” (Interview 1, very low food security). Other coping strategies focused on acquisition of funds or resources needed to secure food. As an example, one participant discussed using research incentives as a source of income to purchase food and other necessities. “Sometimes I wouldn’t eat as much because I knew I wouldn’t be able to do a paid [research] participation experiment until later in the week, so I tried to make the food I have last until then” (Interview 22, low food security).

**TABLE 4:** Participant-identified coping strategies used to improve or maintain food security status.

Coping strategy	Exemplar quotes
Stocking up	“I would get a lot of the pantry items like pasta and cookies [...]. I try to find a lot of things that I can keep in the pantry, just in case of emergencies –Interview 33, very low food security
	“We have like a stock of like instant ramen […] Spam and Vienna sausage so if we ever find ourselves running low on food there's still those things in the pantry just in case.” – Interview 27, marginal food security
Buying in bulk	“We'll buy large quantities of basic foods and will try to make meals from that so we will get canned pumpkins and squashes and make like pumpkin chicken curry and things like that. Because, then you can spread that out, because you make this big quantity and then you can freeze that whole meal and then you can make rice and put it over rice and now that meal lasts forever.” –Interview 40, high food security
	“[We] stock up for like a month's worth of supplies at a time and just use those supplies to its fullest. Once we are out of chicken you switch to only pork, or something until you go through all of that.” – Interview 19, marginal food security
Meal planning	“When I go to the grocery store, I try to shop for like the entire next week ahead, so I don't have to go and get food. So, I’ll think about generally what meals I want to make that week, and I’ll think about if I’m having a busy week, how much frozen food or easy food I should pick up – Interview 3, high food security
	“During the week it was just really tough sometimes to find the time to cook, so I would just set a time on Sunday where I would just cook all of the food that I need. So that way when I come back home from campus, I could just pop it in the microwave and have something to eat.” –Interview 4, high food security
	“I always have to be thinking about food because I’m like, what am I going to eat for breakfast, what am I going to eat for lunch or for dinner, so I have to plan ahead and make sure there is enough [food] and then if there isn't I have to find time to like buy groceries”– Interview 2, very low food security
Cheap groceries	“I'll try to shop at Grocery Outlet instead of Trader Joe's, even though I know that I like the things from Trader Joe's a little bit better. But stores like Grocery Outlets have cheaper foods, even though they're sometimes not the best quality.” – Interview 5, marginal food security
	“I usually like, I check that ads that we get in the mail to see like what's on sale and sometimes I’ll get things there based off of that or like if I’m shopping there like I’ll usually buy the items that are on sale” – Interview 10, marginal food security
Couponing	“I usually try to look for like deal so like on the when I go to target for example, I’ll scan like every single item I have in my cart just to make sure that there aren't any like deals that I’m missing.” – Interview 17, marginal food security
	“I am very anxious; a lot of the time and I always try and coupon and get the cheaper thing and I plan and replan my budget like five times every month” – Interview 20, marginal food security
	“using coupons makes me feel like “oh, I can spend the amount of money on a better thing than I would [normally] get and get more value out of it” than something that is maybe not as nutritious and would be the same price.” – Interview 6, high food security
Research incentives	“I would also supplement my income by doing pay participation research experiments and so I would try to use that money mostly for food and gas.”–Interview 22, low food security
	“I signed up for as many [Research] studies as I could. I think I did like four or five last week […] I was able to get enough food for this week, hopefully for next week as well.” – Interview 33, very low food security
Skipping meals or eating less	“Sometimes I will consciously think about if I have enough money for food and stuff like that, but other times I would forget to eat, and then it would just be on the terms of if hungry, if I’m not hungry I would have a glass of water and like wait for when I should eat or I if forget again.” – Interview 22, low food security
	“In that scenario [Not having money to afford food] I just wouldn't eat since food is such a low priority for me.” –Interview 17, high food security
	“ If I'm tight on money, and you know I don't have that much money to spend on food, then I'll kind of try and eat less that way. My daughter has enough food to eat because I rather obviously go hungry than her. ” –Interview 41, very low food security
	“I was waiting for the instant noodles. The noodles didn't arrive, and I didn’t want to spend extra at the supermarket, so I just decided to skip eating and wait for the second day.” –Interview 15, very low food security
Food from housemates and friends	“My friend group at Davis they're very cognizant of the fact that sometimes like I don't have like a lot of food or like I can't pay for like all the meals and things so they're always down to help” – Interview 27, very low food security
	“I'm not shied to take my friends' food if they don't want to finish it. I had a friend in undergraduate that was just going to toss some cans of food away or some dried pasta, that they didn't really plan on using, so they always just reached out like hey if you don't want it, I’ll take it.” – Interview 38, marginal food security
Events for food	“If I see any kind of food opportunity or something I probably will drop whatever I have and go get it ASAP” – Interview 38, very low food security
	“If I’m ever worried about like food, I would just go on Facebook and start looking around at the free and fair pages and stuff that people are giving out and then that's what I would just go to pick up and get” – Interview 35, high food security
Credit card	“I think having a credit card makes it helpful because then if I need to go over budget it's not like I didn't have the cash on me to do it, or I didn't have the amount in my account to do it” – Interview 39, high food security
Conserving energy	“There are times when I'm so busy and then I don't have anything left in the fridge. I'm like all right, I'm just going to go to sleep. I don't have the energy to go and get it.” – Interview 17, high food security
	“If I had snacks, I'm just like “oh that's a meal that filled me up” and I could sleep... and then I'd be like “oh don't eat dinner” and I’ll just fall asleep and be fine, and then I’ll just eat breakfast when I wake up, kind of just finding ways to conserve how much I would have to eat.” – Interview 1, very low food security

### Food access programs

Within interviews, participants expressed mixed feelings and views on using on-campus, state, and federal food access resources. Most individuals held positive views on food access resources and several individuals stressed the importance of food access resources and benefits associated with use. Although many participants recounted experiences where the resources provided them food in times of need, several participants were reluctant to use food access resources. Reluctant participants and some participants not using food access resources stated that they did not use the resources due to the barriers and stigma associated with use.

#### Benefits to using food access programs

Participants were asked to reflect on current and past food access resource use. Participants who used food access programs recounted improved diet quality, reduced stress related to food procurement, and increased confidence in food acquisition. “I definitely splurge more on things that will make me happy, like snacks. […] [My diet] increased in vegetables and fruits, I feel like those were the pricey options, but having CalFresh [allows me to buy] those healthier options” (Interview 38, marginal food security). One participant recounted how using campus food access programs benefited them. “I feel like when I’m really stressed out, for examinations and stuff, anything I need to do to take care of myself that's [my] last priority like exercising, eating the right foods or even getting food...that's not a worry, but I know I needed to just function as a person. So being able to have something like the pantry […] that really makes the difference in being able to feed myself” (Interview 3, high food security). Participants noted the use of the campus pantry reduced financial burden and allowed for more flexibility in allocating their resources. “If I see that I’m getting near like having to pull from my savings I’ll try getting food from the pantry and consider how I can like make meals based off of [those items]” (Interview 31, marginal food security). Others recounted that the use of food access resources allowed them to improve their diet. “It was a reassurance that [the pantry’s] there for me. It kind of made my day a little bit better because I had those little bits of items I could now plan and look forward to, and to help me to make that basic meal that I have at home a little bit better” (Interview 37, very low food security).

#### Reluctance and barriers to us

Participants who had not used or had discontinued their use of food access resources were asked a series of questions to better understand their decision. One common theme observed among participants, regardless of FSS, was not wanting to take resources away from others with a greater need: “I feel like other people should have them [food access resources], I guess, more than I should” (Interview 33, very low food security). Another student stated, “I've gotten food I think twice or something from the pantry on campus, but I haven't done that too often because sometimes I feel like I don't necessarily need it because I'm not that reliant on it, and I feel like other people might need it more than I do” (Interview 5, marginal food security).

Participants also reported barriers associated with food access programs, such as difficulty accessing on-campus food access resources due to time constraints and limitations of available items. “I think [the pantry’s] a really good resource. I just think the wait times are a bit long, and they do have fresh produce, but the two times I thought about going for it, they ran out before I was able to get there with my schedule” (Interview 20, marginal food security).

## Discussion

The purpose of this study was to investigate knowledge of food insecurity as a concept, evaluate FSS, identify food acquisition-related behaviors, and determine whether behaviors differ according to FSS within college students. Student participants from all food security classification groups were able to define food insecurity. Regardless of FSS, participants recounted similar challenges and barriers associated with food acquisition and reported using a variety of coping strategies. There were also no differences in the number of food access resources used by participants by FSS. From the interviews, a common theme discussed by participants was the fear of taking from others in greater need, regardless of their own needs.

Although purposive sampling efforts were employed, the prevalence of food insecurity in this sample (24%) is substantially lower than the previous estimates reported within University of California students at 42% (35). However, despite most participants being classified as “high” food security by the AFSSM, many participants went on to describe experiences with food insecurity.

Although the AFSSM has been the primary metric used to classify food security in the United States the tool has not been specifically validated among specialized populations, like college students [10,11]. Although the AFSSM remains the gold standard for measuring FSS in the United States, growing evidence suggests it may not adequately capture the unique challenges faced by college students brought on by their enrollment in higher education [7]. When answering food security-related questions, college students may interpret the questions within the AFSSM differently than expected, thus leading to misclassification of FSS [11,52,53]. In a study among college students conducted by Nikolaus et al. [53], cognitive interviews demonstrated that students have varying perceptions resulting in misinterpretation of key terms within the AFSSM, such as “money for more,” “real hunger,” and “balanced meal.” Further, in a mixed-methods study of college students experiencing food insecurity, significant differences were observed between AFSSM categorization and perceived FSS [54]. The findings of this study are consistent with other analyses that demonstrated discrepancies between self-categorized food security and USDA AFSSM classification [54] and with cognitive interviews demonstrating variabilities in student interpretation of the module [53]. This study shows that reliance on food security classification may not identify all college students at risk of experiencing difficulties or anxiety surrounding food access. At the time of publication, no food security measurement tool has been validated for use specifically within the college student population. Accurate assessment is essential for future research and program development. The findings of this study contribute to the previous research supporting the need to develop a valid and reliable food security classification tool for college students [55].

Regardless of FSS, participants face a series of challenges with maintaining food security. Participants recounted experiences in accordance with previous research findings where access [56], finances [35], and time pressures [57] resulted in skipping meals or having to strategize food acquisition. Consistent with the literature, the findings from the current study highlighted transportation and location as barriers that can shape food choices and coping strategies [58,59]. One particular challenge identified among participants was changes in food security and financial security based on the time of the month or year. Participants reported that their diet shifted based on when they received their financial aid, food access benefits, and/or paycheck. Similar observations were reported by Stebleton et al. 2020 [60]., who noted differences in diet and eating patterns based on the time of month or year of student interviews. These reported fluctuations in food security, financial stability, and food availability among the college student population may contribute to varying food insecurity prevalence because the timing of evaluation can yield different results. Future research examining the temporality of food security in college student populations is needed to better understand optimal time frames for retrospective reporting and how to intervene to support students experiencing food insecurity. Beyond the methodological implications, these findings highlight that students face a variety of challenges associated with food security and the need for efforts to mitigate potential challenges.

The results of both the questionnaire and interviews showed that participants from all 4 food security classification groups used a variety of coping strategies to improve or maintain food security. Study results are consistent with previous research findings, which documented the use of coping strategies, such as couponing, buying in bulk, cheap groceries, skipping meals, or eating less [23,25,28]. Additional coping strategies reported within student interviews from the current study included meal planning, using a credit card, and sleeping or reducing activity to conserve energy. Despite their FSS, participants discussed how relying on housemates and friends for food served as a critical coping strategy when food was low or unavailable. The use of this coping strategy has been previously observed in student populations but has been underexplored in individuals classified as having “high” or “marginal” food security [28,57]. When examining motivations for using campus food pantries, previous research observed that participants who were food secure used the pantry as a buffer to ensure adequate funds for other expenses [61]. The use of coping strategies in participants who are not currently classified as food insecure indicates the need to better understand the temporal nature of experiences of food insecurity in college students and how previous experiences with food insecurity may influence food acquisition behaviors and coping strategies used. Further, the use of accepting and sharing food with housemates results in the adoption of household practices that can increase food access and reduce barriers to food adequacy and quality, thereby potentially providing a consistent support system for students regardless of FSS. Consequently, individual food security then becomes reliant on household food security. This phenomenon may underlie challenges for students when answering questions about experiences with individual food security within the AFSSM, potentially resulting in misclassification.

The use of coping strategies within this study, as well as those documented from previous research, demonstrate behaviors attributed to food security have the potential to be overlooked when classifying FSS and measuring the prevalence of food security [27,62]. Within the study population, participants who were marginally food secure used significantly more coping strategies to acquire food than individuals with high food security. Both the current study and previous findings demonstrate that college students and general adult populations with marginal food security face similar experiences and hardships as food insecure individuals [32,[63], [64], [65]]. Thus, these findings suggest that grouping college students classified with marginal food security into a “food secure category” may not be appropriate when examining populations based on a food secure compared with food insecure basis [32,64].

There were no significant differences observed by FSS in the number of food access resources used among the participants in this study. This reinforces that factors beyond need, such as personal beliefs or perceptions, access, and/or awareness, can influence the use of food access resources. Previous research has documented that students facing food insecurity who perceive themselves as being food insecure are more likely to use food access resources and additional coping strategies compared with students facing food insecurity who do not identify as food insecure [54]. Within the present study, participants from all 4 food security classifications who were not using or who had discontinued using on-campus food access resources discussed fears of taking from others with greater need. Stigma and concern of insufficient need as a barrier have also been documented in previous research [66,67]. Results from this study demonstrate a need for improved marketing of on-campus food access resources to promote use. This recommendation is consistent with previous reports, demonstrating a need for positive messaging and a potential rebranding of current food access resources [68].

One particular strength of the present study is the use of a mixed-methods approach to contextualize quantitative findings. In-depth interviews add nuance to questionnaire data, which provides a deeper understanding of college student experiences. The methodology employed included inductive coding to allow for participant experiences to guide the codebook rather than preconceived domains or previously reported themes. Participants were also purposively sampled to represent a variety of viewpoints, because certain student groups may face unique challenges.

No study is without limitations; the present study is less likely to be generalizable due to the sampling methods employed. Despite sampling methods designed to target student populations previously observed to experience a higher prevalence of food insecurity, the prevalence of food insecurity within the sample population was lower than what was observed in weighted estimates from a population-based assessment at the same institution [22]. The institutional setting of this study is also home to robust programming to address food insecurity; thus, these students from this institution may be more knowledgeable about food insecurity as a topic, but their behaviors in response to the experience of food insecurity may differ from students at institutions where food insecurity is not as openly acknowledged or without programs in place to address it. This study was conducted during the spring and summer of 2021 when many students were displaced or relocated due to the COVID-19 pandemic. As a result, food access data and housing arrangements may not have been representative of typical student experiences. Although participants were asked to recall their experiences with food acquisition and related challenges before the COVID-19 pandemic and related shutdowns, recall and recency bias may have been present. Most participants completed their interviews within 7 days of completing their survey, but participant response times varied. As a result, the quantitative data may either capture or omit experiences emphasized in the qualitative findings. The cross–sectional design of the study serves as an additional limitation both in conflation to COVID-19 and in the prevention of drawing relationships between food security and food acquisition behaviors. Further mixed-methods studies should be conducted to investigate the role of coping strategies in food security and help improve food security measurement tools.

### Implications for research and practice

It was observed that students from all food security categorizations used food access resources. Multiple factors, in addition to need, influenced decisions to use food access resources or forego them. This supports the need for universities to offer programs to promote food access resources [69].

Although many components influence food security, students’ perception of their FSS may be a driving factor in identifying their use of coping strategies for food acquisition**.** This study demonstrates that despite being food insecure, participants with low food security employed fewer coping strategies as compared to individuals with very low and marginal food security. Additionally, food access resource use was consistent among all 4 food security classifications. Although the exact reasons for this are unknown, student perceptions of FSS may likely dictate whether students are willing to use these resources compared with those who feel that others have a greater need.

Perceived FSS, in addition to AFSSM food security classification can serve as a potential marker to identify which students are more willing to accept assistance and employ a variety of coping behaviors. However, the findings also suggest that food security classification, according to the USDA AFSSM, may not fully capture anxieties around food acquisition specific to the college student population. Further research is needed to understand the temporality of food insecurity among this population over the course of their academic trajectories and the impact these experiences have on students’ behaviors.

This research also contributes to the growing body of evidence that the USDA AFSSM may not perform as expected in specialized populations, such as college students, and suggests the need to develop specific metrics for identifying FSS in college student populations [11,53,54]. Proper classification of FSS within college students is needed to truly understand the extent and nature of the problem. This understanding can be used to promote the expansion of resources, outreach, and research efforts to ensure these efforts are reaching their target population and mitigating potential health-related consequences associated with food insecurity.

To decrease stigma and barriers, improved marketing efforts among food access resource programs should be implemented to increase use among students. Results from this study can be used to inform the development of better evaluation tools and food access resources in this population. In order to properly measure rates of food insecurity among college students, future tools should include further measures associated with food acquisition, such as stigma, coping strategies, and food access usage. Results from this study can help support marketing efforts among food access programs to increase usage among college students through positive messaging and inclusive language.

## Author contributions

The authors’ responsibilities were as follows **–** ES, GC, RES: designed research; ES, GC: conducted research; ES, GC analyzed data; and ES, GC, RS, LK, DF wrote the paper. ES: had primary responsibility for final content. All authors read and approved the final manuscript.

## Data Availability

Data described in the manuscript, code book, and analytic code will be made available upon request pending application.

## Funding

Funding for the study was provided in part by the University of California Office of the President Global Food Initiative and by the Aggie Compass Basic Needs Center.

## Conflict of interest

The authors report no conflicts of interest.
