# Supplementary material for: Food Acquisition Coping Strategies Vary Based on Food Security Among University Students
Source: Curr Dev Nutr. 2024 Dec 16;9(1):104529. doi: 10.1016/j.cdnut.2024.104529 (PMC11773212; doi:10.1016/j.cdnut.2024.104529)
Supplement: Multimedia component 1 [file mmc1.docx]

**In-depth Interview – Part 1**

Main Survey [asked of all participants]

1. How would you define food insecurity?
   1. How did you come up with this definition?
   2. Follow-up with the USDA definition of Food Insecurity for consistency among participants in Q 2- Q4
2. What circumstances, in your opinion, makes a person food insecure (can be given in list form)?
   1. Why do you believe those situations may cause someone to become food insecure?
3. Based on your experience, have you ever felt food insecure or faced situations in which you were not able to obtain food?
4. How would you define your food security status? (If they having trouble coming up with an answer have them rate on scale of one to ten)
   1. Do you still feel this way?
5. According to your answers to the USDA Food security status module, you are classified as ……….. Do you agree or disagree with assessment?

---------------------------------------------------------------------------------------------------------------------

[If participants indicate that they disagree with the classification, they will answer the following supplemental questions, based upon their answers in the online questionnaire that they filled out prior to taking part in the qualitative portion of this study. If they agree with their classification, the interview will continue with Question 6 :

Why do you disagree with your classification? Let's take a look at your evaluation to determine what answers to the module led to this classification and go through your thought process when you were answering these questions.

Question 1: “(I/We) worried whether (my/our) food would run out before (I/we) got money to buy more.” Was that often true, sometimes true, or never true for (you/your household) in the last 12 months/30 days?

You answered this question __________. Please explain why you answered this question the way that you did. What experiences or circumstances drove you to say __________.

Question 2: “The food that (I/we) bought just didn’t last, and (I/we) didn’t have money to get more.” Was that often, sometimes, or never true for (you/your household) in the last 12 months/30 days?

You answered this question __________. Please explain why you answered this question the way that you did. What experiences or circumstances drove you to say __________.

Question 3: “(I/we) couldn’t afford to eat balanced meals.” Was that often, sometimes, or never true for (you/your household) in the last 12 months/30 days?

You answered this question __________. Please explain why you answered this question the way that you did. What experiences or circumstances drove you to say __________.

Question 4: In the last 12 months/30 days, since last (name of current month), did (you/you or other adults in your household) ever cut the size of your meals or skip meals because there wasn't enough money for food?

You answered this question __________. Please explain why you answered this question the way that you did. What experiences or circumstances drove you to say __________.

Question 5: [IF YES TO QUESTION 4, PLEASE ANSWER THE FOLLOWING] How often did this happen—almost every month/day, some months/weeks but not every month, or in only 1 or 2 months?

You answered this question __________. Please explain why you answered this question the way that you did. What experiences or circumstances drove you to say __________.

Question 6: In the last 12 months/30 days, did you ever eat less than you felt you should because there wasn't enough money for food?

You answered this question __________. Please explain why you answered this question the way that you did. What experiences or circumstances drove you to say __________.

Question 7: In the last 12 months/30 days, were you ever hungry but didn't eat because there wasn't enough money for food?

You answered this question __________. Please explain why you answered this question the way that you did. What experiences or circumstances drove you to say __________.

Question 8: In the last 12 months/30 days, did you lose weight because there wasn't enough money for food?

You answered this question __________. Please explain why you answered this question the way that you did. What experiences or circumstances drove you to say __________.

Question 9: In the last 12 months/30 days, did (you/you or other adults in your household) ever not eat for a whole day because there wasn't enough money for food?

You answered this question __________. Please explain why you answered this question the way that you did. What experiences or circumstances drove you to say __________.

Question 10: [IF YES TO QUESTION 9, PLEASE ANSWER THE FOLLOWING, ASK] How often did this happen—almost every month/day, some months/weeks but not every month/day, or in only 1 or 2 months?

You answered this question __________. Please explain why you answered this question the way that you did. What experiences or circumstances drove you to say __________. ]]

---------------------------------------------------------------------------------------------------------------------

Main Survey [asked of all participants]

1. If you have ever worried about running out of food, what is your thought process; what are your next steps/ how do you cope?

---------------------------------------------------------------------------------------------------------------------

[This section will only be used for participants who have identified as one of the following groups in the online questionnaire that they filled out prior to taking part in the qualitative portion of this study]

Grad students: When answering the UC Davis Food security, stress and covid questionnaire you self-identified as a Graduate student

- How has becoming a graduate student affected the way you answered these questions?
- What circumstances, if any, of being a grad student make these questions difficult to answer?
- Were there any questions missing that would have explained your food security status in more detail?

First Generation College students : When answering the UC Davis Food security, stress and covid questionnaire you self identified as a First Generation College student

- How has becoming a First generation college student affected the way you answered these questions?
- What circumstances, if any, of being a First generation college student make these questions difficult to answer?
- Were there any questions missing that would have explained your food security status in more detail?

Returning/ Non Traditional students: When answering the UC Davis Food security, stress and covid questionnaire you self identified as a Returning/ Non Traditional student.

- How has becoming a Returning/ Non Traditional student affected the way you answered these questions?
- What circumstances, if any, of being a Returning/ Untraditional make these questions difficult to answer?
- Were there any questions missing that would have explained your food security status in more detail?

---------------------------------------------------------------------------------------------------------------------

Main Survey [asked of all participants]

1. In thinking about a typical month, let's walk through the process of obtaining food:
   1. What source of income do you use towards the purchase of food?
   2. The day before you receive this income, what are you eating and where does that food come from?
   3. What prompts you or allows you to purchase food? where do you obtain it?
   4. How do you decide what resources to use to buy food?
   5. What happens if you don’t have enough money to afford the food that you need?

---------------------------------------------------------------------------------------------------------------------

Now, we’re going to talk a little bit more about some of the answers that you gave to the survey that you filled out before this interview.

[This section will only be used for participants who have answered yes to the corresponding question in the online questionnaire that they filled out prior to taking part in the qualitative portion of this study.

In the questionnaire you reported that you have credit card(s)/credit accounts. Does paying off these credit card/credit account balances affect your ability to purchase food?

- - Does having credit cards support you in obtaining food? (ie are you purchasing food on credit?) If so, how so?
  - Do you have an unpaid balance on your credit card due to food purchases?

In the questionnaire you reported that you receive financial assistance, is this your main source of income?

- - Do you use this money to purchase food?
  - If so, how would you cope if you did not have this source of income?

In the questionnaire you reported that you receive financial aid disbursements (how often) ------ do you believe that this affects your food security status. How so?

In the questionnaire you reported that you have one or more unpaid jobs or internships. Does this have an effect on your food security status?

- - If so: Do your eating patterns change based on when you receive your financial aid?

In the questionnaire you reported that you have one or more paid jobs or internships. Does this have an effect on your food security status?

- - Do you use the money that you receive from your job to obtain and purchase food?
  - Is the money that you receive from your job/s the main income you spend on food?
  - How would your food security status be affected if you did not have this income?

You noted that you use on campus food access resources, how has this changed your eating behaviors ?

- - How long have you been using it/them? [How often do you use them?]
  - How do think these have benefited you in the time you have been using it/them
  - Would you be affected if you did not have access to these resources? How so?
  - Beyond the campus resources (like the Pantry and the Compass), are there other coping strategies you have used to get food when you needed it but didn’t have money for it?

You noted that you use CalFresh, how has this changed your eating behaviors ?

- How long have you been using it/them? [How often do you use them?]
- How do think these have benefited you in the time you have been using it/them
- Would you be affected if you did not have access to these resources? How so?

You noted that you do not use any campus or state funded food access resources, is there any reason you do not access these resources?

- Do you think your eating habits/behaviors would change if you had access to food access resources?
- Why does this change your answer?
- Why does this not impact your answer? ]

---------------------------------------------------------------------------------------------------------------------

Main Survey [asked of all participants]

1. Is there anything else that you think we should know about your food security status that we have not already discussed?
